# Validation of a novel prediction model for early mortality in adult trauma patients in three public university hospitals in urban India

**DOI:** 10.1186/s12873-016-0079-0

**Published:** 2016-02-22

**Authors:** Martin Gerdin, Nobhojit Roy, Monty Khajanchi, Vineet Kumar, Li Felländer-Tsai, Max Petzold, Göran Tomson, Johan von Schreeb, On behalf of the Towards Improved Trauma Care Outcomes in India (TITCO)

**Affiliations:** Health Systems and Policy, Department of Public Health Sciences, Karolinska Institutet, Tomtebodavägen 18A, Solna, 171 65 Stockholm, Sweden; Department of Surgery, Bhabha Atomic Research Centre Hospital, Mumbai, India; Tata Institute of Social Sciences, School of Habitat, Mumbai, India; General Surgery, Seth GS Medical College & King Edward Memorial Hospital, Mumbai, India; Department of Surgery, Lokmanya Tilak Municipal Medical College and General Hospital, Mumbai, India; Department of Clinical Science Intervention and Technology, Division of Orthopedics and Biotechnology, Karolinska Institutet, Stockholm, Sweden; Centre for Applied Biostatistics, Occupational and Environmental Medicine, Sahlgrenska Academy, University of Gothenburg, Gothenburg, Sweden; Faculty of Health Sciences, School of Public Health, University of the Witwatersrand, Johannesburg, South Africa; Department of Learning, Informatics, Management and Ethics, Karolinska Institutet, Stockholm, Sweden

**Keywords:** Trauma, Early mortality, India, Prediction models

## Abstract

**Background:**

Trauma is one of the top threats to population health globally. Several prediction models have been developed to supplement clinical judgment in trauma care. Whereas most models have been developed in high-income countries the majority of trauma deaths occur in low- and middle-income countries. Almost 20 % of all global trauma deaths occur in India alone. The aim of this study was to validate a basic clinical prediction model for use in urban Indian university hospitals, and to compare it with existing models for use in early trauma care.

**Methods:**

We conducted a prospective cohort study in three hospitals across urban India. The model we aimed to validate included systolic blood pressure and Glasgow coma scale. We compared this model with three additional models, which all have been designed for use in bedside trauma care, and two single variable models based on systolic blood pressure and Glasgow coma scale respectively. The outcome was early mortality, defined as death within 24 h from the time when vital signs were first measured. We compared the models in terms of discrimination, calibration, and potential clinical consequences using decision curve analysis. Multiple imputation was used to handle missing data. Performance measures are reported using their median and inter-quartile range (IQR) across imputed datasets.

**Results:**

We analysed 4440 patients, out of which 1629 were used as an updating sample and 2811 as a validation sample. We found no evidence that the basic model that included only systolic blood pressure and Glasgow coma scale had worse discrimination or potential clinical consequences compared to the other models. A model that also included heart had better calibration. For the model with systolic blood pressure and Glasgow coma scale the discrimination in terms of area under the receiver operating characteristics curve was 0.846 (IQR 0.841–0.849). Calibration measured by estimating a calibration slope was 1.183 (IQR 1.168–1.202). Decision curve analysis revealed that using this model could potentially result in 45 fewer unnecessary surveys per 100 patients.

**Conclusions:**

A basic clinical prediction model with only two parameters may prove to be a feasible alternative to more complex models in contexts such as the Indian public university hospitals studied here. We present a colour-coded chart to further simplify the decision making in early trauma care.

## Background

Trauma is a major threat to global population health, accounting for more deaths than HIV/AIDS, tuberculosis, malaria, and maternal conditions, combined [1]. The outcomes of trauma patient are highly dependent on early appropriate treatment decisions. Therefore numerous clinical prediction models have been developed to aid and support clinicians in early trauma care [2–10]. In medicine, clinical prediction models are used to estimate an individual patient’s risk of experiencing a specific outcome, often by combining several patient level parameters in a multivariate model [11, 12]. Clinical prediction models are intended to supplement, not replace, clinical judgement by functioning as decision-support tools [13, 14].

In trauma care prediction models are used for example to identify patients that need to be referred to a trauma centre or taken to the intensive care unit. They are also used to inform on prognosis. Most models designed for use in trauma care have been developed for specific trauma patient subgroups, for example patients with traumatic brain injury, rather than a general trauma population [15]. Furthermore, several models include parameters not always known on initial presentation in the emergency room, such as age or injury severity score [8–10, 16]. Also, models include categorised continuous predictors, for example systolic blood pressure [8–10, 16], an approach likely to reduce predictive potential [17].

The majority of clinical prediction models for trauma care come from high-income countries, whereas over 90 % of deaths occur in low- and middle-income countries [18–20]. India, a lower middle-income country accounts for about 20 % of all global trauma deaths [21]. A significant part of these deaths occur in hospitals. In an attempt to contribute to reduced trauma mortality in Indian hospitals we recently derived a prediction model using data from three public university hospitals in urban India [22]. The basis for this model was that it should serve all types of trauma patients, be as simple as possible to be potentially used bedside, and only include vital signs routinely collected on arrival. The model included only systolic blood pressure and Glasgow coma scale and the aim of this study was to validate this model, and to compare it with existing models for use in early trauma care. We also compared the performance of the model with systolic blood pressure and Glasgow coma scale to the predictive performance of these two vital signs on their own.

## Methods

### Study design and context

We conducted a temporal validation study as part of a larger prospective multi-centre observational cohort project called Towards Improved Trauma care Outcomes in India (TITCO). Data from three hospitals were analysed. Jai Prakash Narayan Apex Trauma Center (JPNATC), All India Institute of Medical Sciences, New Delhi, has about 180 beds and is a dedicated trauma centre. Lokmanya Tilak Municipal General Hospital (LTMGH), Mumbai is a public university hospital. It hosts a dedicated trauma ward with 14 beds. The Institute of Post-Graduate Medical Education and Research and Seth Sukhlal Karnani Memorial Hospital (IPGMER & SSKM), Kolkata is also a public university hospital, but lacks a dedicated area for receiving and managing trauma patients. All included hospitals are referral centres, and hence a large proportion of patients are transferred from other hospitals. Prehospital triage is generally not performed in any of these cities.

### Models

We recently derived a model for predicting in-hospital mortality in adult trauma patients within 24 h of the first recorded vital signs [22]. This model included only systolic blood pressure and Glasgow coma scale. In the same study we also assessed a model that in addition to systolic blood pressure and Glasgow coma scale included heart rate. These three variables were chosen a priori because they were the most commonly used in the study sites. Both systolic blood pressure and heart rate were modelled using restricted cubic splines to handle potential non-linear associations between these variables and early mortality log odds. In the derivation study we found no significant differences in predictive performance measures between these two models. The study included 1629 patients aged ≥15 years with history of trauma arriving at the three centres described above between October 1, 2013 and January 11, 2014.

We choose to compare the two models outlined above with models recently published by Kondo et al.[10] and Perel et al.[8] The model published by Kondo et al. includes Glasgow coma scale, age, and systolic blood pressure. Perel et al. published one comprehensive model, also available as an online calculator, and a simple model available as a colour chart. Like Kondo’s model, Perel’s simple model included systolic blood pressure, age, and Glasgow coma scale. Our rationale for using the Kondo and Perel models as comparisons was firstly that both have been presented as simply models that can be used bedside. Secondly, in contrast to most other established models neither include respiratory rate as a predictor as respiratory rate has been repeatedly shown to be poorly collected even in high-income contexts [23, 24].

Kondo’s model was derived and validated using 27 154 patients included in the Japan Trauma Data Bank between 2004 and 2009. The primary outcome was death before discharge. Perel’s model was derived using data from 20 127 patients included in the Clinical Randomisation of an Antifibrinolytic in Significant Haemorrhage (CRASH-2) trial, which involved 274 hospitals in 40 countries [25]. The model was then validated using data from 14 220 patients from Trauma Audit and Research Network (TARN). The TARN database primarily includes patients seen in centres in England and Wales. The primary outcome was death within 28 days of injury. A detailed account of the Kondo and Perel models are available in their respective original publications.

Finally, we compared the predictive performance of our model that included systolic blood pressure and Glasgow coma scale and each of these two vital signs when used on their own. This analysis was done to assess the predictive performance of an even simpler approach then that suggested by our two variable model.

### Data

In each of the hospitals in this study one data collector collected data for 8 h per day through direct observation of patient resuscitation in the emergency room and data extraction from patient files. All data collected was part of routine data collection. The data collector’s role was to systematically note them down as close to patient arrival as possible. The data collector worked day, evening, and night duties according to a rotating schedule so that all possible shifts were covered during the course of a month. For patients admitted outside of the data collector’s shift, data were extracted from patient files. Data collectors followed up on patients for the first 24 h after admittance, or until discharge or death. Data were uploaded on a weekly basis and project management had weekly data review meetings. Two on-site quality control sessions were performed during which a random selection of 1–5 % of entries were cross-checked with official patient records. No major deviations were observed.

### Eligibility criteria

Patients aged ≥15 years were included if they presented with history of trauma and were admitted or died between arrival and admission. Patients with isolated limb injury, i.e., isolated extremity fractures without vascular injury, and patients who were dead on arrival were not included. Patients with isolated extremity fractures are at the studied centres not part of the general trauma care pathway but attended to by orthopaedic surgeons alone.

### Sample size considerations

We calculated the sample size to enable detection of a decrease in model discrimination of 0.05 percentage points with 80 % power, compared to discrimination in the derivation sample. According to simulation studies, this required an effective sample size of 200 patients with the primary outcome, also referred to as events [13, 26]. Hence, the first 200 consecutive events and all non-events, i.e., patients who survived or were discharged alive before the first 24 h, occurring during the same time period as the events were included. These parameters corresponded to all patients enrolled between January 12, 2014 and July 23, 2014.

### Variables

The outcome was early mortality, defined as in-hospital mortality within 24 h of the time when the first vital signs were recorded after patient arrival at the study centres. Five variables were required to apply the four models: systolic blood pressure (mmHg), heart rate (beats per minute), Glasgow coma scale and age (years).

### Missing data strategy

We used multiple imputation using chained equations to handle missing data to maximise efficiency under the assumption that data was missing at random. Patterns of “missingness” and variable distributions were explored before the imputation model was finalised. Early mortality was included in the imputation model. Each hospital was imputed separately, using the same imputation model, and thereafter combined into a single dataset. The number of imputed datasets generated was equal to the percentage of incomplete observations in the hospital with the highest percentage of incomplete observations [27].

### Statistical methods and analyses

We used Stata (Stata: Release 13. StataCorp LP, College Station, Texas) for statistical analyses. A significance level of 5 % was used. We calculated 95 % confidence intervals or the inter quartile range (IQR), defined as the range between the 25th and 75th percentiles, as applicable. Descriptive statistics were used to analyse and present sample characteristics. We performed tests individually in each imputed dataset and present the results using the median P-value and IQR across imputed datasets. A median P-value < 0.05 was deemed as significant.

#### Step 1: Updating

**Fig. 1:**
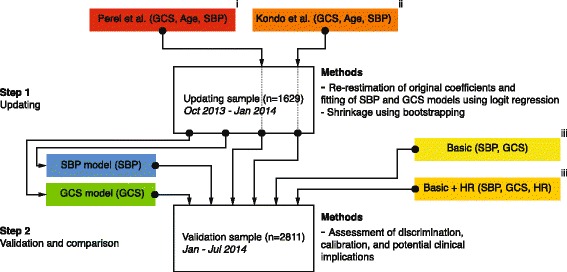
Flow chart outlining the analysis process. In step 1, the models by Kondo et al. and Perel et al. was updated by re-estimation of the original coefficients using logit regression. Two simple models, one based only on systolic blood pressure and one based only on Glasgow coma scale, was fitted using logit regression. The basic model with systolic blood pressure and Glasgow coma scale and the basic model + heart rate were not updated in step 1, as they were derived in a previously published study using the same sample. In step 2, the basic model and the basic model + heart rate, the updated Kondo and Perel models, and the fitted systolic blood pressure and Glasgow coma scale models were validated and compared in a temporally independent sample compared to the updating sample. Both the updating and the validation samples were from the same three hospitals. i [8], ii [10], iii [22]. Abbreviations: GCS Glasgow Coma Scale, HR Heart Rate, Jan January, Jul July, Oct October, SBP Systolic Blood Pressure

We conducted our analyses in two steps (Fig. 1). First, we updated the Kondo and Perel models using the sample used to derive our basic model with systolic blood pressure and Glasgow coma scale, and the basic model + heart rate [22]. This sample is henceforth referred to as the updating sample. The updating sample was also used to fit two simple logit models, one with only Glasgow coma scale and one with only systolic blood pressure. The latter was modelled using restricted cubic splines with four knots placed at equally spaced percentiles. The rationale for updating the Kondo and Perel models before comparing them with our models was that we aimed to make the comparison as unbiased as possible. A valid critique of clinical prediction model studies is that many studies present new models without considering existing evidence [28]. Simple updating methods have been proposed as means to incorporate existing evidence by adjusting available models to a new context.

We used a re-estimation method to update the Kondo and Perel models, meaning that for each model we fitted a logit model that included the original parameters (for the Perel model we excluded a parameter indicating whether the patient received tranexamic acid and a random intercept) [28]. Parameters were kept in the model regardless of their statistical significance upon updating. To avoid overfitting these models we repeated the estimation process in 300 bootstrap samples drawn with replacement. Each bootstrap model was then used to generate a linear predictor in the original, not bootstrapped, updating sample. This linear predictor was used in a logit model with early mortality as the dependent variable. We then calculated a linear shrinkage factor as the mean of the coefficients of the all linear predictors from each of the 300 bootstrap models. The same approach was applied to the systolic blood pressure and the Glasgow coma scale model. This linear shrinkage factor was applied to the final Kondo, Perel, systolic blood pressure, and Glasgow coma scale coefficients.

#### Step 2: Validation and comparison

Once we had updated the Kondo and Perel and fitted the systolic blood pressure and Glasgow coma scale models in the updating sample we fitted each of the six models, i.e., our basic model with systolic blood pressure and Glasgow coma scale, our basic model + heart rate, the Kondo, the Perel model, the systolic blood pressure model, and the Glasgow coma scale model in the validation sample. We then assessed and compared the discrimination, calibration, and net benefit of the basic model with each of the other models. We measured discrimination using the area under the receiver operating characteristics curve (AUROCC), sensitivity, and specificity. The AUROCC is a measure that ranges between 0 and 1, where 1 indicates perfect discrimination and 0.5 a discrimination as good as chance. We assessed calibration visually using a calibration plot and statistically by estimating the calibration slope.

The net benefit was estimated using decision curve analysis, a method proposed as means to quantify the clinical implications of using a prediction model [29–31]. The starting point of decision curve analysis is a clinical scenario. We choose a scenario in which there are several trauma patients in the trauma receiving area that have been surveyed. In monitoring the patients, the clinician may choose to do a repeated survey of everyone, survey no one, or to use a prediction model to decide whom to survey. The net benefit at a specific probability of early mortality, called threshold probability, can be used to estimate the number of patients identified as in need of a repeated survey, without the clinician having to do any unnecessary surveys. This would be a crucial advantage in context where resources are limited.

### Ethical considerations

Ethical clearance was obtained from each of the participating hospitals. The names of the ethical bodies and reference numbers were Institute Ethics Committee All India Institute of Medical Sciences (EC/NP-279/2013 RP-Ol/2013), Ethics Committee of the Staff and Research Society (IEC/11/13), and the Institute of Post-Graduate Medical Education and Research Research Oversight Committee (IEC/279) for Jai Prakash Narayan Apex Trauma Center, Lokmanya Tilak Municipal General Hospital, and the Institute of Post-Graduate Medical Education and Research and Seth Sukhlal Karnani Memorial Hospital respectively. All review boards granted the study a waiver of informed consent. We applied for a waiver of informed consent because the patients included in this study often arrived to hospital with an altered level of consciousness and in severe physical and psychological distress, and because this study only involved the collection of routine data and did not alter the care provided in any way.

## Results

We analysed data from 1629 patients for the updating of the Kondo and Perel models and the fitting of the systolic blood pressure and Glasgow coma scale and data from 2811 patients to validate and compare all six models (Table 1). In the updating sample the highest percentage of incomplete observations was 51 % and hence 51 imputed datasets were generated. For the validation sample corresponding figures were 33 % and 33 imputed datasets. The updating and validation samples were similar with regards to age, proportion of male patients, time between injury and arrival at the study centres, proportion of patients transferred from other centres, mechanism of injury, as well as initial vital signs. Early mortality was 6 % in the updating sample and 7 % in the validation sample.

**Table 1:** Sample characteristics

Variable	Updating cohort (*n* = 1629)	Validation cohort (*n* = 2811)	Fraction of missing data (%)
Age	35 (24–47)	35 (25–46)	0/0
Male %	80 (78–82)	80 (79–82)	0/0
Mechanism of injury %			0/0
Fall	27 (25–29)	26 (25–28)	
Railway accident	7 (6–8)	6 (5–7)	
Road traffic accident	46 (44–49)	46 (44–47)	
Assault	9 (7–10)	11 (9–12)	
Burn	6 (5–7)	7 (6–8)	
Other	4 (3–5)	5 (4–5)	
Delay, time from injury to admission (hours)	7 (2–28)	7 (2–26)	6/4
Transferred %	68 (65–70)	71 (69–72)	0/0
Systolic blood pressure	116 (106–125)	120 (110–130)	20/9
Heart rate	90 (80–98)	89 (80–98)	18/3
Glasgow coma scale	15 (9–15)	15 (9–15)	20/8
24-h mortality %	6 (4–7)	7 (6–8)	0/0

Data is presented as medians with inter quartile range or percentages with 95 % confidence intervals as applicable

### Step 1: Updating

All parameters included in the original Kondo et al. model were significant in the updating sample except for age < 60 years (P-value 0.525) (Tables 2 and 3). The median AUROCC in the updating sample was 0.808 (IQR 0.801–0.816) and the median calibration slope was 1.046 (IQR 1.022–1.090) (Table 4). In other words, Kondo’s model discriminated adequately between survivors and non-survivors and was well calibrated in the updating sample. The shrinkage factor was 0.987, indicating little need for shrinkage before applying this updated model to a new sample.

**Table 2:** Outline of models

Model	Parameter	β^a^ (SE)
Basic^b^	SBP, SBF 1	−0.026 (0.006)
	SBP, SBF 2	−0.044 (0.036)
	SBP, SBF 3	0.419 (0.244)
	GCS	−0.228 (0.031)
	Intercept	2.302 (0.577)
Basic + HR^b^	SBP, SBF 1	−0.024 (0.008)
	SBP, SBF 2	−0.042 (0.038)
	SBP, SBF 3	0.378 (0.256)
	HR, SBF 1	−0.003 (0.013)
	HR, SBF 2	−0.018 (0.121)
	HR, SBF 3	0.225 (0.559)
	GCS	−0.229 (0.032)
	Intercept	2.214 (0.662)
Kondo et al.	GCS	−0.254 (0.030)
	Age < 60 years	−0.225 (0.354)
	SBP < 60	0^c^
	60 ≤ SBP ≤ 120	−2.067 (0.525)
	SBP > 120	−2.511 (0.585)
	Intercept	1.919 (0.616)
Perel et al.^d^	Age	−0.067 (0.142)
	Age^2^	0.002 (0.003)
	Age^3^	−1.3·10^-5^ (<0.001)
	SBP	0.019 (0.024)
	SBP^2^	−0.001 (<0.001)
	SBP^3^	2.3·10^-6^ (<0.001)
	GCS	−0.679 (0.188)
	GCS^2^	0.024 (0.010)
	Intercept	3.678 (2.016)
Systolic blood pressure model	SBP, SBF 1	−0.285 (0.030)
	SBP, SBF 2	−0.079 (0.247)
	SBP, SBF 3	−0.031 (0.006)
	Intercept	−0.052 (0.033)
Glasgow coma scale model	GCS	0.533 (0.222)
	Intercept	0.595 (0.460)

Coefficients (β) of all models were estimated in the updating sample

^a^After shrinkage

^b^As reported in the derivation study [22]. In the basic and basic + HR models systolic blood pressure and heart rate were modelled using restricted cubic splines. ^c^Reference. ^d^Age and systolic blood pressure were modelled using cubic terms whereas Glasgow coma scale was modelled using a quadratic term. Abbreviations: *GCS* Glasgow coma scale, *HR* heart rate, *SBP* systolic blood pressure, *SBF* spline basis function

**Table 3:** Estimated probability of early mortality per model

Model	Updating sample	Validation sample
Basic	0.02 (0.01–0.05)	0.01 (0.01–0.04)
Basic + HR	0.02 (0.01–0.05)	0.01 (0.01–0.04)
Kondo et al.	0.02 (0.02–0.06)	0.02 (0.01–0.07)
Perel et al.	0.02 (0.01–0.04)	0.02 (0.01–0.04)
Systolic blood pressure model	0.04 (0.02–0.06)	0.02 (0.02–0.04)
Glasgow coma scale model	0.01 (0.01–0.07)	0.01 (0.01–0.07)

Model estimates are presented as median probabilities of early mortality with inter quartile ranges. The basic model included only systolic blood pressure and Glasgow coma scale [22]. The basic + HR model included heart rate in addition to systolic blood pressure and Glasgow coma scale [22]. Kondo’s model included Glasgow coma scale, age, and systolic blood pressure [10], as did Perel’s model [8]. *Abbreviations*: *HR* heart rate

**Table 4:** Selected model performance measures

Model	Updating sample			Validation sample	
	SF	AUROCC (IQR)	CS (IQR)	AUROCC (IQR)	CS (IQR)
Basic	0.927	0.845 (0.841–0.854)	0.997 (0.955–1.017)	0.846 (0.841–0.849)	1.183 (1.168–1.202)
Basic + HR	0.896	0.848 (0.842–0.857)	0.985 (0.957–1.020)	0.846 (0.842–0.850)	1.126 (1.105–1.142)
Kondo et al.	0.987	0.815 (0.808–0.822)	1.112 (1.062–1.146)	0.842 (0.838–0.846)	1.480 (1.458–1.497)
Perel et al.	0.960	0.844 (0.835–0.853)	1.010 (0.978–1.048)	0.847 (0.844–0.851)	1.192 (1.178–1.215)
SBP	1.011	0.780 (0.772–0.788)	1.258 (1.185–1.280)	0.681 (0.676–0.688)	0.795 (0.765–0.819)
GCS	0.969	0.802 (0.795–0.807)	1.198 (1.166–1.231)	0.838 (0.835–0.842)	1.476 (1.449–1.490)

*Abbreviations*: *AUROCC* area under the receiver operating characteristics curve, *CS* calibration slope, *GCS* Glasgow coma scale, *HR* heart rate, *IQR* inter-quartile range, *SBP* systolic blood pressure, *SF* shrinkage factor

Perel et al. modelled age as a cubic term. However, age was still not significant in the updating sample (P-value 0.768, joint test of all three coefficients being simultaneously equal to 0). Both systolic blood pressure, modelled as a cubic term, and Glasgow coma scale, modelled as a quadratic term, were significant. The median AUROCC in the updating sample was 0.844 (IQR 0.835–0.853) and the median calibration slope was 1.010 (IQR 0.978–1.048). As with Kondo’s model, these figures indicate that Perel’s model discriminated and calibrated well in the updating sample. The shrinkage factor was 0.960.

For the systolic blood pressure model we fitted a systolic blood pressure model using restricted cubic splines with four knots. The median AUROCC in the updating sample was 0.780 (IQR 0.772–0.788) and the median calibration slope was 1.258 (IQR 1.185–1.280). The shrinkage factor was 0.969. For the Glasgow coma scale model we fitted Glasgow coma scale as a linear term. Median AUROCC was 0.802 (IQR 0.795–0.807), median calibration slope was 1.198 (IQR 1.166–1.231) and the shrinkage factor was 1.011.

### Step 2: Validation and comparison

**Fig. 2:**
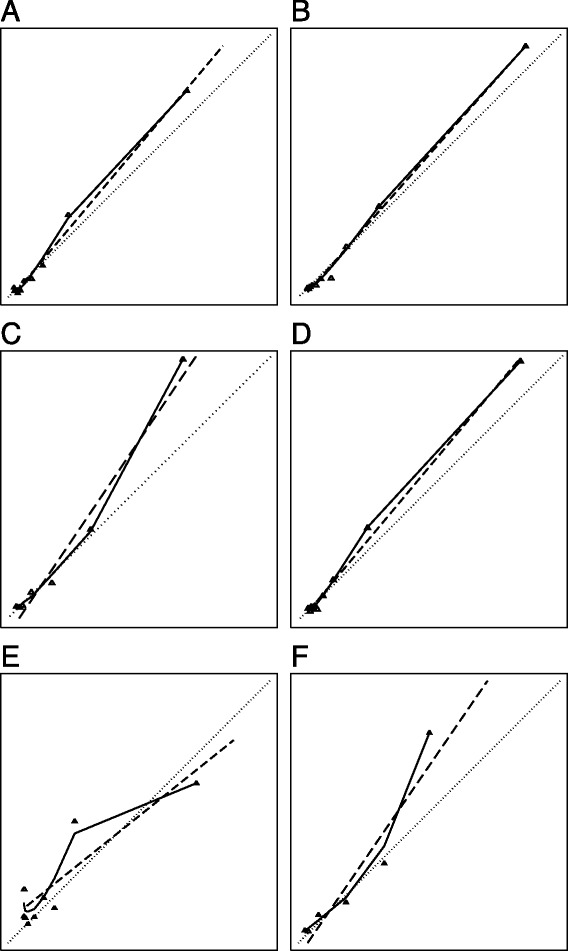
**a**–**d**. Calibration plots. On the x-axis is predicted probability of early mortality and on the y-axis is observed probability of early mortality across ten quantiles of predicted early mortality. The dotted line indicates perfect calibration. The solid line indicates a smoothed association between observed and predicted probability of early mortality and the dashed line a graphical representation of the calibration slope. **a** Basic model with systolic blood pressure and Glasgow coma scale [22] **b** Basic model + heart rate [22] **c** Model by Kondo et al.[10] **d** Model by Perel et al.[8] **e** Systolic blood pressure model (**f**) Glasgow coma scale model

When applied to a temporally independent sample the basic model that included only systolic blood pressure and Glasgow coma scale had a median AUROCC of 0.846 (IQR 0.841–0.849) and a median calibration slope of 1.183 (IQR 1.168–1.202) (Table 4) (Fig. 2). The basic model + heart rate had a median AUROCC of 0.846 (IQR 0.842–0.850) and a median calibration slope of 1.126 (IQR 1.105–1.142). Kondo’s model had a median AUROCC of 0.840 (IQR 0.836–0.844) and a median calibration slope of 1.327 (IQR 1.310–1.346) whereas corresponding figures for Perel’s model were 0.847 (IQR 0.844–0.851) and 1.192 (IQR 1.178–1.215). Hence, these four models both discriminated and calibrated well when applied to the validation sample.

In contrast, the systolic blood pressure model had a median AUROCC of 0.681 (IQR 0.676–0.688) and the median calibration slope was 0.795 (IQR 0.765–0.819). For the Glasgow coma scale model the median AUROCC was 0.838 (IQR 0.835–0.842) and the median calibration slope was 1.476 (IQR 1.449–1.490). These numbers indicate that the systolic blood pressure model had poor discrimination and overestimated the risk of early mortality in the validation sample, whereas the Glasgow coma scale model discriminated adequately but underestimated the risk of early mortality in the validation sample. However, a visual inspection of the corresponding calibration plots indicates that for the systolic blood pressure model the slope misrepresent the calibration pattern (Fig. 2e). This pattern can be better described as if the model underestimated the risk for patients with low and medium risk, whereas it overestimates the risk in patients with high risk.

The AUROCC of the basic model with systolic blood pressure and Glasgow coma scale was not significantly different to that of the basic model + heart rate (median *P*-value 0.701, IQR 0.587–0.830), Kondo’s model (median *P*-value 0.557, IQR 0.321–0.764), Perel’s model (median *P*-value 0.707, IQR 0.313–0.840), or the Glasgow coma scale model (median P-value 0.359, IQR 0.223–0.533). The AUROCC of the basic model was significantly better than that of the systolic blood pressure model (median *P*-value <0.001, IQR 0.000–0.000).

The calibration slope of the basic model was significantly worse compared to the calibration slope of the basic model with heart rate (median *P*-value < 0.001, IQR 0.000–0.000). On the other hand it was significantly better compared to Kondo’s model, the systolic blood pressure model, and the Glasgow coma scale model (all median *P*-values < 0.001, IQR 0.000–0.000). The calibration slope of the basic model was not significantly different compared to that of Perel’s model (median *P*-value 0.200, IQR 0.054–0.519).

**Fig. 3:**
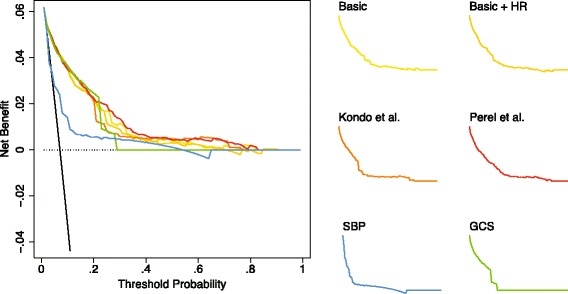
Decision curves associated with each model. The curves to the right are the same as in the left graph, but separated to better visualise and help compare curve shape. The basic model included only systolic blood pressure and Glasgow coma scale [22]. The basic + HR model included heart rate in addition to systolic blood pressure and Glasgow coma scale [22]. Kondo’s model included Glasgow coma scale, age, and systolic blood pressure [10], as did Perel’s model [8]. The systolic blood pressure model included only systolic blood pressure and the Glasgow coma scale model included only Glasgow coma scale. Abbreviations: GCS Glasgow Coma Scale, HR Heart Rate, SBP Systolic Blood Pressure

With regards to the potential clinical implications of applying these models the decision curve analysis revealed very similar curves for the basic model, the basic model with heart rate, Kondo’s model, and the Perel’s model (Fig. 3). In interpreting these curves, we see that using any of the models tested here is superior to the option of surveying all patients in the range of predicted probabilities of early mortality 0.05–0.2. Using 0.05 as the threshold probability we found that the median net benefit was 0.045 (IQR 0.045–0.046) for the basic model, 0.045 (IQR 0.045–0.045) for the basic model with heart rate, 0.045 (IQR 0.045–0.045) for Kondo’s model, and 0.046 (IQR 0.045–0.046) for Perel’s model.

These figures can be interpreted as using any of these models result in a net of five true positives per 100 patients without increasing the number of false positives, or compared to using no model 45 fewer false positives per 100 patients. In other words, using a model to decide whom to do a repeated survey on instead of surveying everyone would lead to 45 fewer unnecessary surveys per 100 patients. In contrast, the curves for the systolic blood pressure model and the Glasgow coma scale model deviate substantially from those of the four other models, indicating less positive clinical implications.

## Discussion

This study indicated that systolic blood pressure and Glasgow coma scale could be enough to predict early mortality in adult trauma patients in three public university hospitals in urban India. We found no evidence that the models published by Kondo and Perel performed significantly better in terms of discrimination, calibration, or potential clinical implications compared to a model that only included systolic blood pressure and Glasgow coma scale. In contrast to other models a model with only systolic blood pressure and Glasgow coma scale does not require the clinician to know variables such as age [8-10]. This constitutes an important advantage, as such variables are not always available to trauma care providers, especially in the early management phases. Adding heart rate to the basic model did significantly improve calibration, but not discrimination or potential clinical implications.

It is interesting to note that although age is generally considered to be an important predictor of trauma mortality it was not significant when we re-estimated the coefficients of Kondo’s and Perel’s models in our updating sample. Kondo et al. included age as an indicator variable, where patients aged < 60 years had a lower odds of early mortality compared to patients aged ≥ 60 years. In contrast, Perel et al. included age as a cubic term, indicating a higher risk at young and old ages. The reason why age failed to prove significant in our study may be that the patients in our cohorts were substantially younger compared to the patients studied by both Kondo and Perel. We argue that although age definitely influences mortality risk and is an important epidemiological tool the usefulness of such models is limited for example in the not too uncommon scenario where the patient is unconscious and there is no relative present. When this happens, models that rely only on vital signs can still be applied.

**Fig. 4:**
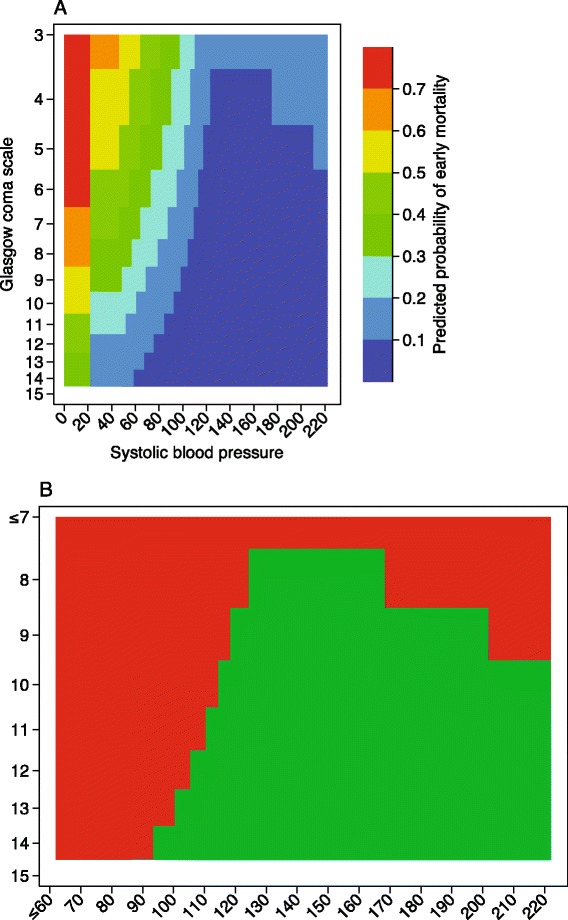
**a**, **b**. Simple colour coded charts for obtaining a predicted probability and a triage category based on the basic model with systolic blood pressure and Glasgow coma scale. **b** The cutoff is at a predicted probability of 0.05

Hence, we argue that there is scope for a new, simpler, model that relies only on routinely measured vital signs. A common criticism of prediction model studies is that they do not clearly explain how to use the model to estimate prognosis [2]. Using only two predictors allowed us to create a coded chart that can be used to obtain the predicted probability of early mortality (Fig. 4a, b). Provided appropriate cut-offs are identified, the chart can be used to identify patients in need of a repeated survey. Compared to more complex models this chart can be used by anyone in the trauma receiving area. For the studied context this was deemed essential.

It was interesting to note the differences between the two, and three variable models compared to the models based on singly vital signs. Even though both the systolic blood pressure and the Glasgow coma scale model discriminated on pair with other published models, calibration was worse compared to the multivariable models assessed in this study. Furthermore, the decision curve analysis clearly shows that single parameter models do not add much in comparison to the survey all or non options, and hence fare substantially worse than the multivariable models in this aspect.

Finally, patients generally arrived late, with a median time from injury to arrival at the study centres of seven hours. This is substantially longer compared to other studies, for example Perel et al. reported a median time from injury to arrival of two hours [8]. The long time between injury and arrival to the study centres observed here is likely a result of patients being taken to other hospitals first, before they are transferred to a higher level of care. In fact, around 70 % of patients included in this study were transferred from other hospitals. Earlier research from this context indicates that little to no treatment is initiated in the pre-hospital setting, at least in Mumbai.

Various reasons for transferring patients have been reported, including lack of space, beds, and the relevant clinical specialities [32]. Hence, we studied a mix of direct admission and transferred patients. It was a conscious decision to include a heterogeneous trauma population as we wanted a model applicable to any trauma patient arriving. In comparison, many established models are developed for a specific trauma population subgroup, such as patients sustaining road traffic injury, or patients with traumatic bleeding or traumatic brain injury [7, 8, 16].

### Methodological considerations

First, it may be argued that our findings are specific to the three hospitals included and that the generalisability of these findings is limited. To some extent this claim is true. We have not studied external validity in terms of performance in different settings. Thus, we do not claim that the model works in other contexts, for example private setups or in high-income countries. However, we would argue that many urban university hospitals at least in India share the characteristics of the hospitals in this study and that our findings may be transferred to such centres.

Next, it is likely that using broad inclusion criteria had a negative impact on the model’s performance. However, from a clinical perspective and as outlined above broad inclusion criteria may strengthen the model’s usefulness as it can be applied to any trauma patient that arrives to the studied hospitals. Finally, we used early mortality as the outcome. Late mortality, functional outcomes, and quality of life are important trauma care end-points and should be further studied in the future to fully understand the outcome of trauma care.

## Conclusions

We found that a prediction model with only systolic blood pressure and Glasgow coma scale predicted early mortality in adult trauma patients in three public university hospitals in urban India. This model had adequate discrimination, calibration, as well as potential clinical implications. In contexts where the availability of skilled staff is limited, most patients are transferred from other centres, and the trauma patients presenting constitute a heterogeneous population this simplified model may prove useful in triaging patients for initial survey. Because it can be used without performing any calculation it offers an important advantage to models that include more variables. Furthermore, as it only includes vital signs it does not require care providers to know patient age or injury mechanism, and may hence be used early in the management and on unconscious patients.

## Abbreviations

AUROCC: area under the receiver operating characteristics curve
CRASH: clinical randomisation of an antifibrinolytic in significant haemorrhage
IPGMER: The Institute of Post-Graduate Medical Education and Research
IQR: inter-quartile range
JPNATC: Jai Prakash Narayan Apex Trauma Center
LTMGH: Lokmanya Tilak Municipal General Hospital
SSKM: Seth Sukhlal Karnani Memorial Hospital
TARN: Trauma Audit and Research Network
TITCO: towards improved trauma care in India
TRIPOD: transparent reporting of a multivariable prediction model for individual prognosis or diagnosis
